# Rituximab Safety Profile: A Seven-Year Retrospective Analysis From Southern India

**DOI:** 10.7759/cureus.74454

**Published:** 2024-11-25

**Authors:** Lakshmi Jayasree, Princy L Palatty, Laxmi Govindraj, Gokul A Anand, Gokul B Dev, Bharath Shabu, Tinu T S, Abhishek A Nair

**Affiliations:** 1 Pharmacology, Amrita School of Medicine, Amrita Institute of Medical Sciences, Amrita Vishwa Vidyapeetham, Ernakulam, IND; 2 Pharmacovigilance, Regional Training Center and ADR (Adverse Drug Reaction) Monitoring Center, Amrita Institute of Medical Science, Amrita Vishwa Vidyapeetham, Ernakulam, IND; 3 Pharmacovigilance, Pharmacovigilance Programme of India, Indian Pharmacopoeia Commission, Ghaziabad, IND

**Keywords:** adverse drug reactions, causality assessment, infusion related reactions, modified hartwig siegel scale, pharmacovigilance, rituximab, rituximab-induced lung disease, safety profile, who-umc criteria

## Abstract

Objective: This study aimed to analyze the pattern, severity, and outcomes of adverse drug reactions (ADRs) associated with rituximab use reported to a regional pharmacovigilance center in Kerala, India.

Methods: This retrospective study analyzed rituximab-associated ADRs reported from 2017 to 2023. ADRs were assessed using the WHO-UMC criteria for causality and the Modified Hartwig Siegel Scale for severity.

Results: A total of 74 patients reported ADRs, with 49 being female. The majority (27 patients) were in the 51- to 60-year age group. Malignancies accounted for 58 cases of rituximab use. Most ADRs (69 cases) occurred with the first dose, with infusion reactions being the most common. Causality assessment revealed that 48 ADRs were probably related to rituximab. Severity analysis using the Modified Hartwig Siegel Scale showed that 67 ADRs were level two (mild), while two were level seven (severe). The majority of patients (72) recovered. However, rituximab-induced lung disease, observed in two cases, was associated with higher mortality.

Conclusion: Rituximab demonstrated an acceptable safety profile, with most ADRs being non-serious and manageable. The high recovery rate reflects effective ADR management. However, the potential for serious complications, particularly rituximab-induced lung disease, highlights the need for vigilant monitoring, especially during the first infusions. These findings enhance understanding of rituximab’s real-world safety profile and underscore the importance of standardized protocols for ADR mitigation.

## Introduction

Rituximab, a chimeric monoclonal antibody targeting CD20-positive B cells, has revolutionized the treatment of numerous hematological and autoimmune disorders since its introduction in 1997 [1]. Initially approved for non-Hodgkin's lymphoma, its therapeutic applications have significantly expanded, becoming integral in the management of chronic lymphocytic leukemia, rheumatoid arthritis, and antineutrophilic cytoplasmic antibody (ANCA)-associated vasculitis [2-4].

Rituximab’s B-cell depletion mechanism has also prompted its exploration in various off-label indications, such as immune thrombocytopenic purpura (ITP), Sjögren’s syndrome (SS), cryoglobulinemia, pemphigus, membranous nephropathy, and other refractory autoimmune diseases, highlighting its versatility [5]. However, this widespread use necessitates vigilant safety monitoring [6]. Despite its efficacy across multiple disorders, rituximab is associated with a spectrum of adverse drug reactions (ADRs) [7].

ADRs linked to rituximab range from common infusion-related reactions (IRRs) to rarer but serious complications [6]. IRRs are the most frequently observed ADRs, especially during the first administration, occurring in up to 77% of patients. These reactions typically arise within 30-120 minutes of starting the infusion and present as symptoms such as fever, chills, rigors, flushing, nausea, pruritus, and headache. Severe symptoms, though less common, may include bronchospasm, hypotension, and angioedema. The underlying mechanism is primarily attributed to cytokine release syndrome caused by the rapid destruction of B cells. Management strategies for IRRs involve premedication with antipyretics, antihistamines, and occasionally corticosteroids, as well as temporary interruption or slowing of the infusion rate [8,9].

Beyond IRRs, rituximab is associated with more severe ADRs that can significantly impact patient outcomes. These include hematological toxicities such as neutropenia (including late-onset), thrombocytopenia, and hypogammaglobulinemia. Patients receiving rituximab are also at an increased risk of bacterial and viral infections, particularly Hepatitis B virus (HBV) reactivation. Other significant ADRs include skin reactions ranging from rash to rare cases of Stevens-Johnson syndrome and toxic epidermal necrolysis, pulmonary complications like interstitial lung disease and pneumonitis, and neurological conditions such as progressive multifocal leukoencephalopathy. Cardiac issues, serum sickness, and gastrointestinal perforations, though less common, have also been reported [10].

The severity of these potential ADRs is underscored by the U.S. Food and Drug Administration's black box warning [11]. This warning highlights four critical safety concerns: fatal infusion reactions (approximately 80% occurring during the first infusion), severe mucocutaneous reactions (some with fatal outcomes), HBV reactivation (which can result in fulminant hepatitis, hepatic failure, and death), and progressive multifocal leukoencephalopathy, often fatal [11]. These warnings emphasize the necessity of vigilant patient monitoring during rituximab therapy [12].

Understanding rituximab's safety profile is essential to optimizing its therapeutic use, as ADRs may require treatment modifications or discontinuation [13]. Moreover, potential regional variations in ADR patterns, influenced by genetic, environmental, or healthcare system factors, highlight the need for locale-specific safety data [14].

This study aimed to analyze rituximab-associated ADRs reported to a regional pharmacovigilance center in Kerala, India. It examined the nature of these ADRs, their causal relationship to rituximab, their severity, and patient outcomes. By enhancing the understanding of rituximab's safety profile, this analysis seeks to inform local clinical practice and contribute to the global knowledge base on rituximab-associated ADRs across diverse populations [15,16].

This article was previously presented as an oral presentation at the 8th Annual Sri Ramachandra Pharmacology Insight and Rapid Review Course - ASPIRE 2024, held from January 25 to 29, 2024, where it received the best oral presentation award.

## Materials and methods

Study design and setting

We conducted a retrospective descriptive study analyzing suspected ADRs to rituximab reported to the Regional Training Centre and ADR Monitoring Centre of Pharmacovigilance at Amrita Institute of Medical Sciences, Kerala, India, from 2017 to 2023. The study aimed to provide an overview of rituximab-associated ADRs in this region. Ethical approval was obtained from the Ethics Committee of Amrita School of Medicine (ECASM-AIMS-2024-403) prior to data collection.

Data collection

A structured data collection process was employed to ensure comprehensive and accurate information gathering. The pharmacovigilance database was systematically reviewed to identify all cases of suspected ADRs associated with rituximab during the study period. Cases were traced back to patients using the records from the Regional Training Centre of Pharmacovigilance at Amrita Institute of Medical Sciences, Kochi. Relevant data were extracted from electronic medical records following the case record form designed for this study. A waiver of consent was granted by the Institutional Ethics Committee, as the study relied on retrospective data.

Data extraction

For each identified case, information was extracted on patient demographics, including age, gender, and relevant medical history, as well as rituximab treatment details such as indication for use, dosage, frequency, and duration. Data on concomitant medications, including the name, dosage, and duration of other drugs used concurrently, were collected. Details of ADRs, including descriptions of the adverse event, onset time, duration, and outcomes, were also recorded. Relevant laboratory values at the time of ADR occurrence and information on the management of ADRs, including interventions such as drug discontinuation or dose modification, were obtained.

Assessment tools

Three standardized tools were used to assess various aspects of the reported ADRs. The WHO-UMC Causality Assessment Tool determined the likelihood of a causal relationship between rituximab and the reported ADRs. The Modified Hartwig Siegel Scale for Severity Assessment categorized the severity of ADRs into seven levels. Additionally, the Common Terminology Criteria for Adverse Events (CTCAE) version 5.0 systematically graded the severity of adverse events, ensuring consistency across cases.

Data analysis

Descriptive statistical analysis was performed using IBM SPSS Statistics for Windows, Version 23 (Released 2015; IBM Corp., Armonk, New York). Categorical data were summarized as frequencies and percentages, while quantitative data were presented as means and standard deviations. Chi-square tests and Fisher's exact tests were applied to compare categorical data between groups.

## Results

**Figure 1:**
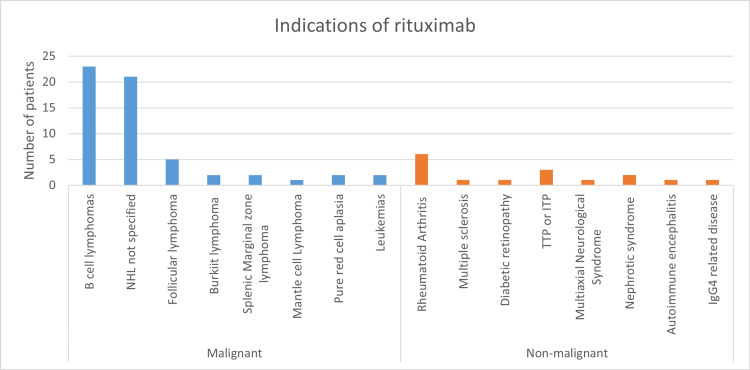
Indications of rituximab in the study population. NHL: non-Hodgkins lymphoma, TTP: thrombotic thrombocytopenic purpura, ITP: immune thrombocytopenic purpura.

This retrospective study analyzed ADRs associated with rituximab in 74 patients. This constituted 2.47 % of all the ADRs reported to our center during the period 2017 to 2023. Female patients predominated 49 (66%), with the majority in the 51- to 60-year age group 27 (36.5%). Malignancies accounted for 58 (78.4%) of rituximab indications (Table 1, Figure 1)

**Table 1:** Baseline demographic details (N=74).

Baseline demographics	Categories	Number (%)
Age categories(years)	21 to 30	5 (6.8%)
	31 to 40	5 (6.8%)
	41 to 50	15 (20.3%)
	51 to 60	27 (36.5%)
	61 to 70	13 (17.6%)
	71 to 80	8 (10.8%)
	Above 80	1 (1.4%)
Gender	Female	49 (66%)
	Male	25 (34%)
Indication	Malignant	58 (78.4%)
	Non-malignant	16 (22%)

ADR characterization showed a predominance of Type B (idiosyncratic) reactions, accounting for 59 cases (79.7%). The majority of ADRs, 69 cases (93.2%), occurred during the first dose administration. Most patients, 72 (97.3%), recovered, while 2 (2.7%) experienced fatal outcomes (Table 2).

**Table 2:** Details of adverse drug reactions (ADRs) (N=74).

Features	Categories	Number of ADRs (%)
Type of ADR	Type A (augmented) reactions	13 (17.6%)
	Type B (idiosyncratic) reactions	59 (79.7%)
	Type C (chronic) reactions	2 (2.7%)
Time of occurrence of ADR	First dose	69 (93.2%)
	Second dose	3 (4.1%)
	Sixth dose	2 (2.7%)
Outcome of ADR at the time of reporting	Fatal	2 (2.7%)
	Recovered	72 (97.3%)
Causality assessment	Certain	5 (6.8%)
	Probable	48 (64.9%)
	Possible	21 (28.4%)

Detailed descriptions of the ADRs by type are given in Table 3.

**Table 3:** Details of symptoms of adverse drug reactions (ADRs) by type.

Type of ADR	Presenting symptoms	Number of patients
Type A	Upper respiratory tract symptoms	4
	Mucositis	3
	Nausea/vomiting	3
	Neutropenia	2
	Constipation	1
Type B	Infusion reactions	49
	Breathing difficulty/chest discomfort	17
	Itching	14
	Shivering	10
	Throat irritation	4
	Others	4
	Anaphylaxis	9
	Breathing difficulty/chest discomfort/wheeze	7
	Not specified	2
	Others	1
Type C	Rituximab-Induced Lung disease	2

**Figure 2:**
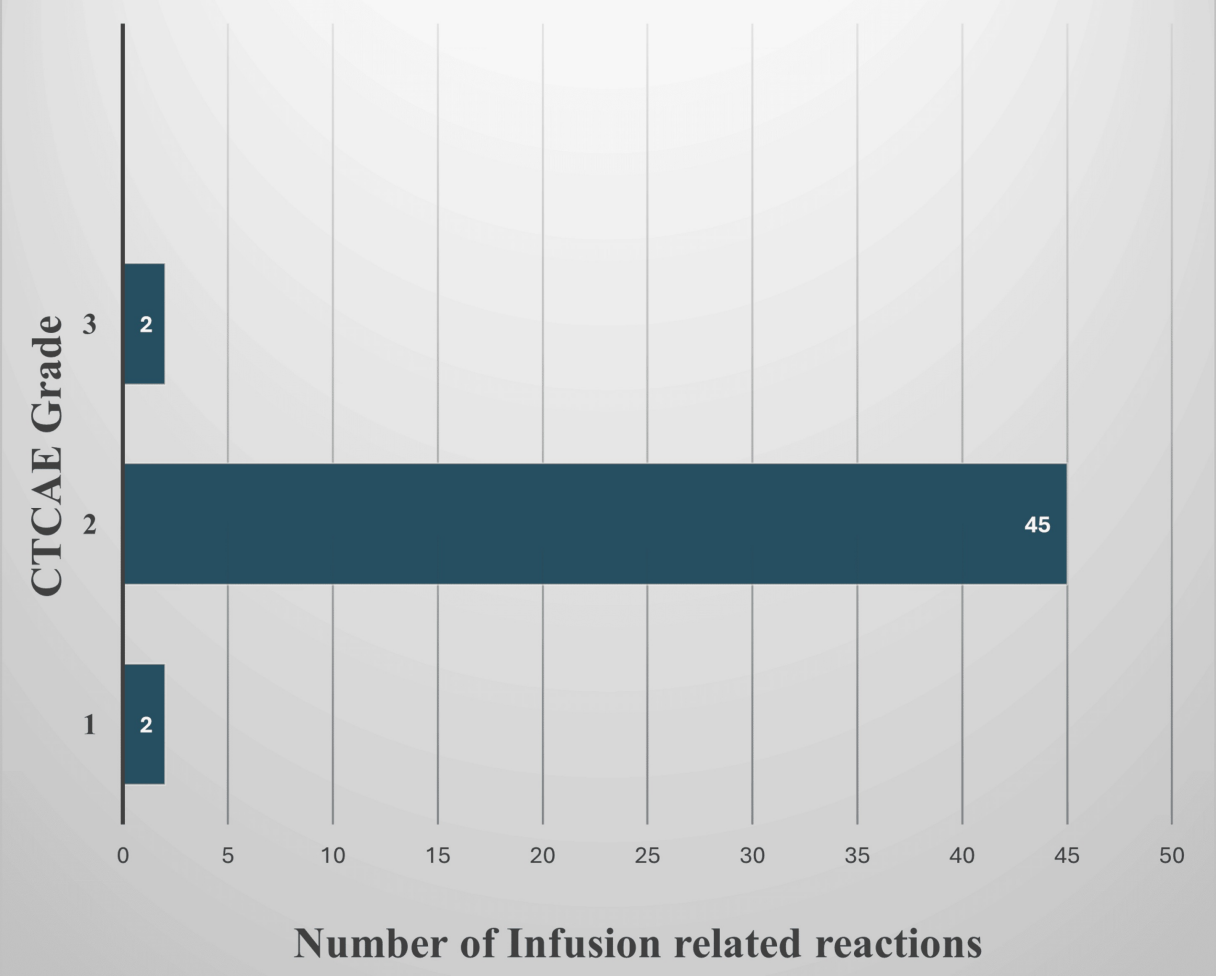
Infusion-related reactions graded according to Common Terminology Criteria for Adverse Events (CTCAE) version 5.0 (N=49).

Infusion-related reactions were the most frequently observed type of ADR. According to the CTCAE version 5.0, the majority of these reactions, 45 (91.8%), were classified as Grade 2 (Figure 2). Grade 1 reactions included two patients who developed a sore throat after the infusion was completed, which did not require interruption of the infusion. Two patients experienced persistent chest discomfort despite initial symptomatic treatment and infusion interruption, leading to classification as Grade 3. All other infusion-related reactions (Table 3) resolved with infusion interruption and symptomatic management, and were therefore classified as Grade 2.

**Figure 3:**
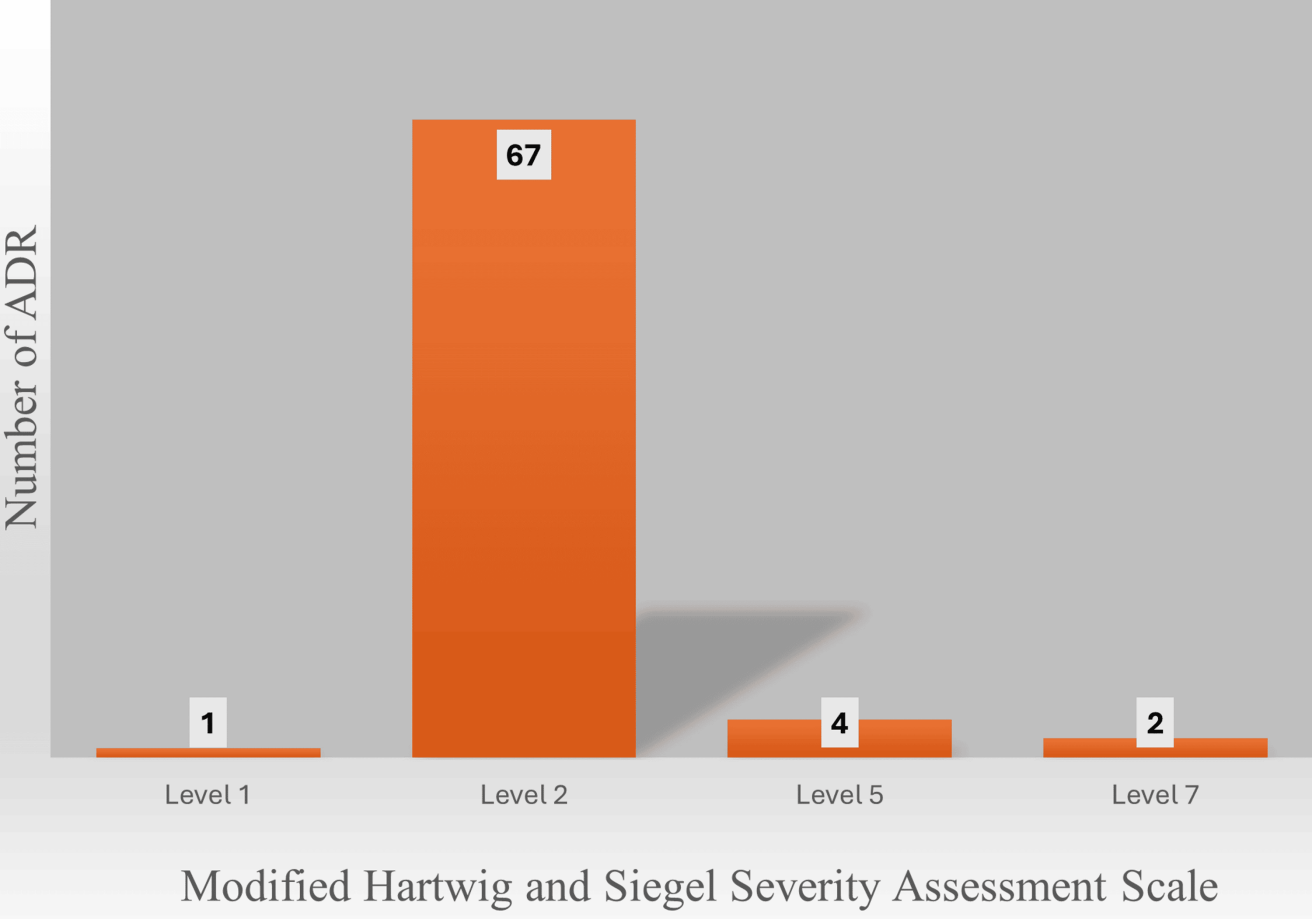
Severity of adverse drug reactions (ADR) using Modified Hartwig Siegel Scale for severity assessment (N=74).

Additionally, anaphylaxis was observed in 9 patients (12.2%). Severity analysis, conducted using the Modified Hartwig and Siegel Severity Assessment Scale, showed that the majority of ADRs, 67 cases (90.5%), were classified as Level 2 (Figure 3).

**Figure 4:**
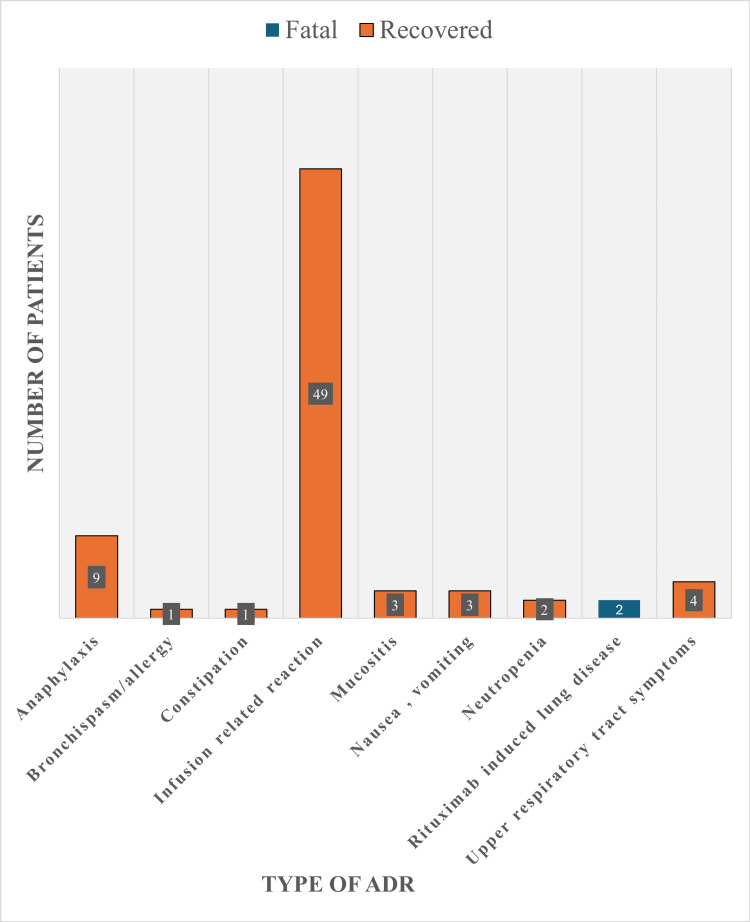
Statistically significant association (p < 0.001) between adverse drug reaction (ADR) types and patient outcomes in rituximab treatment.

Figure 4 illustrates the distribution and outcomes of various ADRs. Infusion-related reactions were the most common, occurring in 49 cases (66.2%), followed by anaphylaxis in 9 cases (12.2%). Most ADR types, including these frequent ones, had 100% recovery rates. Rituximab-induced lung disease (R-ILD) was observed in 2 cases (2.7%). A statistically significant association was identified between ADR types and patient outcomes (p < 0.001).

## Discussion

Our study provides valuable insights into the safety profile of rituximab in a real-world setting, encompassing both malignant and non-malignant indications. The findings indicate that while rituximab is associated with a range of ADRs, most are non-serious and manageable, supporting its continued use in therapy. The predominance of ADRs during the first dose, observed in 69 cases (93.2%) in our study, aligns with findings from Vogel et al., who reported that infusion-related reactions occur primarily during the first infusion and decrease in frequency with subsequent administrations [8]. This underscores the importance of vigilant monitoring during initial treatments, particularly given our observation that infusion-related reactions were the most common ADR, occurring in 49 cases (66.2%).

The breakdown of ADR types revealed a predominance of Type B (idiosyncratic) reactions, accounting for 59 cases (79.7%), followed by Type A (augmented) reactions in 13 cases (17.6%) and Type C (chronic) reactions in 2 cases (2.7%). This distribution, with a high proportion of Type B reactions, is consistent with rituximab's known safety profile, where infusion-related reactions and hypersensitivity are common [8]. However, our proportion of Type B reactions is higher than some reports in the literature. Kasi et al., in their review of rituximab-associated adverse events, noted significant occurrences of both immediate (Type B) and delayed (Type A) reactions [6]. The higher proportion of Type B reactions in our study may be attributed to the diversity of our patient population, the wide range of indications for rituximab use, and the study's focus on capturing immediate and short-term ADRs.

Notably, we observed a high recovery rate of 72 cases (97.3%), with a fatality rate of 2 cases (2.7%). This recovery rate is comparable to that reported by van Vollenhoven et al. in their long-term safety analysis of rituximab in rheumatoid arthritis, where serious adverse events rarely led to treatment discontinuation [7].

As infusion-related reactions are known to occur with rituximab, premedication is followed in all cases according to standard guidelines. Patients receiving intravenous rituximab are pre-medicated with paracetamol, diphenhydramine, and steroids (for longer infusion times). Despite this, 66.2% of the reported ADRs in our study were infusion-related reactions, aligning with the findings of Kasi et al., who reported a similarly high incidence of these reactions [6]. However, we noted a higher rate of anaphylaxis in nine cases (12.2%) compared to other studies, which have reported rates ranging from 5% to 7% [17, 18]. This discrepancy warrants further investigation and may highlight the need for heightened awareness and preparedness for severe allergic reactions during rituximab administration. While we observed cases of mucositis in three patients (4.1%), it is important to note that these patients were also receiving cyclophosphamide, a known causative agent for this ADR [19]. Therefore, mucositis cannot be attributed solely to rituximab. This finding highlights the complexity of ADR causality assessments in patients undergoing combination therapies.

Our analysis revealed a statistically significant association between ADR types and patient outcomes (p < 0.001), suggesting that the nature of the ADR may be predictive of clinical course and resolution. This finding aligns with observations by Kasi et al. in their systematic review, where certain ADR types, particularly infusion-related reactions, were associated with specific clinical outcomes [6].

R-ILD, observed in two (2.7%) of our cases, represents a rare but significant complication. This aligns with the 0.01-10% incidence reported in the literature [20]. R-ILD's pathogenesis remains unclear, with presentations varying from acute organizing pneumonia to indolent interstitial patterns [20, 21]. Diagnosis typically combines clinical, radiological, and occasionally histopathological findings, with management usually involving rituximab discontinuation and corticosteroid therapy [21]. Instances of R-ILD were characterized by acute and severe clinical manifestations in both cases. These patients, aged 46 and 54 years, developed significant respiratory distress after four to five cycles of rituximab treatment for non-Hodgkin's lymphoma. Both cases rapidly progressed to fatal outcomes within one to two weeks of symptom onset, despite interventions including drug withdrawal and supportive care. Chest imaging revealed interstitial lung disease patterns. These findings align with literature reports of acute-onset R-ILD, such as Wagner et al., who described acute interstitial pneumonitis occurring within days to weeks of rituximab administration [21]. The timing after multiple cycles is consistent with observations by Liu et al., that R-ILD can occur after any treatment cycle [22]. Our cases’ progression timeline is more consistent with literature reports; for instance, Alexandrescu et al. noted a median time from symptom onset to death of 13 days in fatal cases [23]. The uniformly acute and severe presentation in our cases contrasts with the wider spectrum of presentations, from indolent to acute, described by Lioté et al. [24]. These findings highlight the potential for severe R-ILD presentations, emphasizing the need for heightened vigilance, prompt intervention, and further research into predictive factors and management strategies for severe R-ILD, particularly in patients presenting with acute respiratory symptoms during or shortly after rituximab treatment.

Causality assessment found 48 (64.9%) of ADRs to be probable, which is significantly higher than the 26.9% reported by Mohebbi et al. [25]. This notable difference could be attributed to several factors, including variations in patient populations, differences in reporting practices, or potential variations in the application of causality assessment criteria. The higher rate of probable causality underscores the importance of vigilant monitoring and prompt management of ADRs in patients receiving rituximab across various indications.

The severity distribution in our study, using the Modified Hartwig and Siegel Severity Assessment Scale, revealed that the majority of ADRs, 67 (90.5%), were classified as Level 2, indicating mild reactions requiring only temporary discontinuation of the drug or prolongation of hospitalization by less than one day. A small proportion of cases were categorized as Level 1 (1.4%), Level 5 (5.4%), and Level 7 (2.7%), representing very mild, life-threatening, and fatal reactions, respectively. This distribution aligns with findings from larger studies, such as the one by van Vollenhoven et al. in their long-term safety analysis of rituximab in rheumatoid arthritis, where most adverse events were mild to moderate [7]. However, our study observed a higher rate of severe reactions compared to some reports. The presence of life-threatening and fatal outcomes in our cohort, albeit rare, underscores the need for vigilant monitoring and preparedness for managing severe complications throughout the course of rituximab therapy. This is particularly crucial given the diverse indications for which rituximab is used, as highlighted by Salles et al. [12].

Demystifying rituximab's adverse reaction profile from this study supports its increased and continued use in therapy. The predominance of Level 2 ADRs (Modified Hartwig Siegel Scale) indicates that most adverse reactions are manageable. Consequently, rituximab can be prescribed without undue hesitancy to achieve therapeutic outcomes, provided appropriate precautions and monitoring are implemented.

However, it is important to acknowledge that the observed ADR profile may be influenced by reporting gaps and underreporting phenomena, which are common challenges in pharmacovigilance studies. Healthcare providers may be more inclined to report serious or unexpected reactions, while mild to moderate ADRs might go undocumented. This underreporting bias could result in an underestimation of the true frequency of Level 1 and Level 2 ADRs. Therefore, while our findings support rituximab's manageable safety profile, the actual incidence of mild to moderate adverse reactions could be higher than reported.

Recommendation

Rituximab is known for its potential to cause ADRs, particularly during the first dose and in infusion-related contexts. Based on this study, we recommend premedicating patients receiving rituximab as a precautionary measure. This approach aims to reduce the risk and severity of ADRs, particularly in high-risk groups such as females and patients aged 51-60 years. The premedication protocol, which includes glucocorticoids and antihistamines, should be standardized yet tailored to individual patient characteristics, potentially improving rituximab's overall safety profile.

Limitations

The retrospective nature of this study and the potential for underreporting of ADRs are limitations that were considered during data interpretation. However, the use of standardized assessment tools strengthens the reliability of our findings. Future prospective studies could provide more comprehensive data on the long-term safety profile of rituximab.

## Conclusions

This real-world assessment of the safety profile of rituximab across both malignant and non-malignant conditions provides valuable insights to guide its optimal use in clinical practice. The findings demonstrate that the majority of ADRs associated with rituximab are non-serious and can be effectively managed with appropriate interventions.

A key observation was the predominance of Type B (idiosyncratic) reactions, particularly infusion-related events occurring predominantly with the first dose administration. These reactions were observed despite premedication, underscoring the critical importance of vigilant patient monitoring during initial treatment cycles to promptly identify and mitigate such events. Notably, the study documented a high overall recovery rate, with adverse events rarely leading to treatment discontinuation, findings aligned with long-term safety data.

In addition, the observation of rare but severe complications, such as rituximab-induced interstitial lung disease, highlights the necessity of prompt recognition and management of severe ADRs throughout the course of therapy.

Importantly, the higher rate of "probable" causality assessments in this study, compared to prior work, underscores the value of comprehensive ADR reporting and attribution. This is particularly relevant given the expanding clinical applications of rituximab. Strengthening pharmacovigilance activities in real-world healthcare settings will enhance understanding of the risks associated with rituximab in relation to its substantial therapeutic benefits.

In conclusion, this study reinforces the overall manageable ADR profile of rituximab while emphasizing the need for heightened vigilance, optimization of premedication practices, and prompt recognition and management of rare but serious complications. These findings provide valuable guidance for clinicians, patients, and policymakers in navigating the risk-benefit considerations of this critical therapeutic agent, ultimately ensuring its safe and effective utilization to maximize patient outcomes.
